# Supplementary material for: A Circulating Risk Score, Based on Combined Expression of Exo-miR-130a-3p and Fibrinopeptide A, as Predictive Biomarker of Relapse in Resectable Non-Small Cell Lung Cancer Patients
Source: Cancers (Basel). 2022 Jul 14;14(14):3412. doi: 10.3390/cancers14143412 (PMC9317031; doi:10.3390/cancers14143412)
Supplement: Supplementary file 1 [file cancers-14-03412-s001.zip › cancers-1673210-supplementary_SC.pdf]

Supplementary Materials: A Circulating Risk Score, Based on Combined Expression of Exo-miR-130a-3p and Fibrinopeptide A, as Predictive Biomarker of Relapse in Resectable Non-Small Cell Lung Cancer Patients

Silvia Marconi, Michela Croce, Giovanna Chiorino, Giovanni Rossi, Francesca Guana, Aldo Profumo, Paola Ostano, Angela Alama, Luca Longo, De Luca Giuseppa, Mariella Dono, Maria Giovanna Dal Bello, Marco Ponassi, Camillo Rosano, Paolo Romano, Zita Cavalieri, Massimiliano Grassi, Marco Tagliamento, Lodovica Zullo, Consuelo Venturi, Chiara Dellepiane, Luca Mastracci, Elisa Bennicelli, Paolo Pronzato, Carlo Genova and Simona Coco

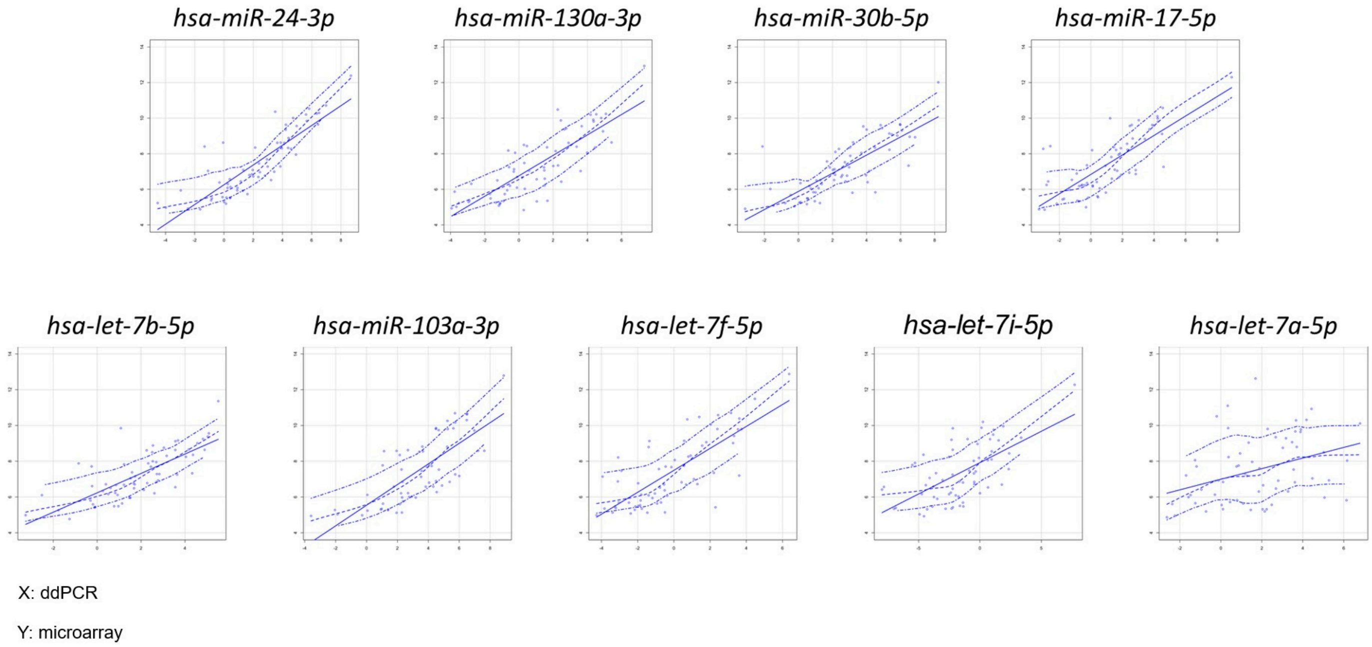

Figure S1. Scatter plots of miRNA's expression values comparing ddPCR (x-axis) and microarray (y-axis) data.

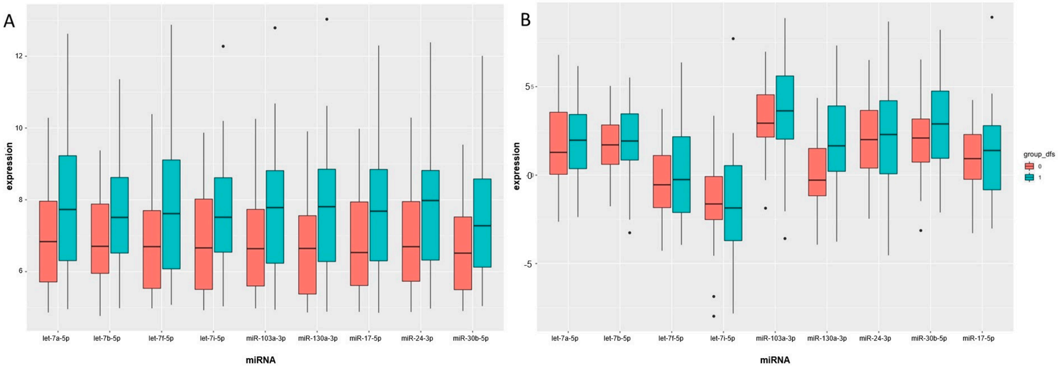

Figure S2. Box plots of 9 Exo-miR expression levels obtained by microarray (**A**) and ddPCR (**B**). Red and blue boxplots are related to the patient without and with event, respectively.

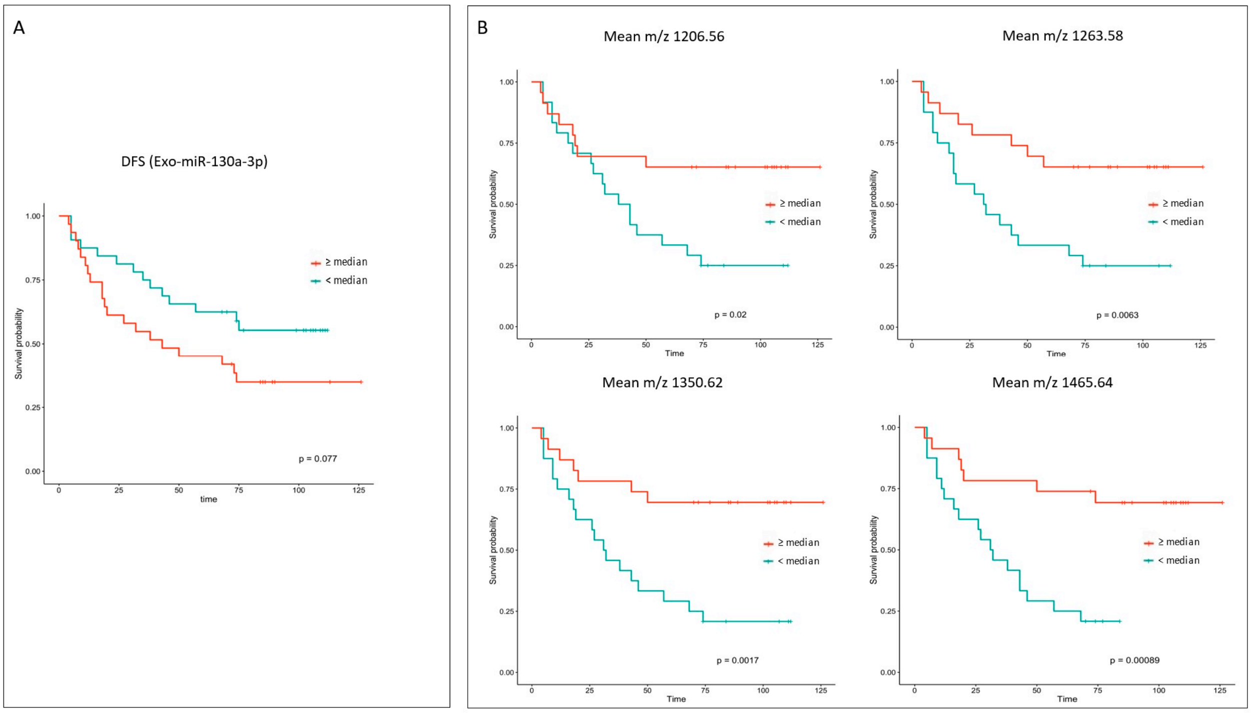

Figure S3. (**A**) Kaplan–Meier curves obtained for Exo-miR130a-3p by stratifying 63 patients according to the median value of its expression in ddPCR; (**B**) Kaplan–Meier curves obtained for the 4 FpA-derived fragments by stratifying 47 patients according to the median value of their signals.
